# Suspected Spontaneous Reports of Birth Defects in the UK Associated with the Use of Carbimazole and Propylthiouracil in Pregnancy

**DOI:** 10.4061/2011/235130

**Published:** 2011-09-13

**Authors:** Pamela Bowman, Bijay Vaidya

**Affiliations:** ^1^Department of Paediatrics, Royal Devon and Exeter Hospital, Exeter, Devon EX25DW, UK; ^2^Department of Endocrinology, Royal Devon and Exeter Hospital, Exeter, Devon EX25DW, UK; ^3^Peninsula NIHR Clinical Research Facility, Level 2, Peninsula Medical School, University of Exeter, Barrack Road, Exeter, Devon EX25DW, UK

## Abstract

The concept of a carbimazole embryopathy underlies current Endocrine Society advice to avoid this drug in early pregnancy, favouring propylthiouracil as an alternative for the treatment of maternal hyperthyroidism. We aimed to establish whether suspected spontaneous reporting of adverse drug reactions in the UK via the Yellow Card Scheme supports a carbimazole embryopathy and the lack of association between propylthiouracil and congenital anomalies. All birth defects related to maternal treatment with carbimazole or propylthiouracil reported over a 47-year period via the Yellow Card Scheme were analysed. 57 cases with 97 anomalies were reported following in utero exposure to carbimazole. These anomalies included aplasia cutis, choanal atresia, tracheo-oesophageal fistula, and patent vitellointestinal duct, which have previously been reported in association with carbimazole/methimazole exposure in utero. Only 6 cases with 11 anomalies were reported for propylthiouracil, all within the last 15 years. Therefore, these findings may support a carbimazole embryopathy. There are few birth defects associated with propylthiouracil, but this should be interpreted in the context of higher historical prescription rates for carbimazole.

## 1. Introduction

Hyperthyroidism, primarily caused by Graves' disease, affects about 1 in 500 pregnancies. Although not common, it is important to recognise and treat maternal hyperthyroidism, because failing to do so can have detrimental effects. In the mother, untreated hyperthyroidism can cause spontaneous miscarriage, pregnancy induced hypertension, preterm labour, congestive cardiac failure, and thyroid storm; for the fetus this could mean still birth, intrauterine growth restriction, or low birth-weight [1]. Further, hyperthyroid states in the mother have been associated with congenital anomalies including oesophageal atresia, tracheo-oesophageal fistula, and biliary tree atresia [2, 3].

There is also an association between the antithyroid drugs used to treat maternal hyperthyroidism and congenital anomalies. This association is most widely reported for carbimazole and its active metabolite, methimazole, such that the concept of a carbimazole embryopathy is being increasingly acknowledged amongst prescribing clinicians [4–9]. There has been no convincing link between the alternative thionamide drug propylthiouracil and birth defects [10] despite the rate of placental transfer of the drug being the same as that of carbimazole [11]. Both drugs are equally efficacious at controlling maternal hyperthyroidism [12]. This has led to the Endocrine Society's current advice to use propylthiouracil as a first-line drug during pregnancy, if available, especially during first trimester organogenesis [13]. Carbimazole or methimazole should be used only if propylthiouracil is not available or if the patient cannot tolerate or has an adverse response to it [13].

Recognition of serious adverse effects of anti-thyroid drugs in pregnancy is dependent upon reporting of such effects by prescribing clinicians. Since 1964, the Yellow Card Scheme has allowed healthcare professionals involved in prescribing in the UK to report suspected serious adverse drug reactions (ADRs) to the Commission on Human Medicines (CHM)/Medicines and Healthcare Products Regulatory Agency (MHRA). The professional reporting the suspected ADR submits a Yellow Card found at the back of the British National Formulary, or electronically via the MHRA website, giving brief clinical details supporting their suspicions that the drug is responsible for the adverse outcome(s) seen. In addition, pharmaceutical companies are legally required to report suspected serious ADRs of their products. Since October 2005, patients have also been able to report suspected ADRs through the Yellow Card Scheme.

In this paper, we aimed to establish whether spontaneous reporting via the Yellow Card Scheme in the UK lends support to an association between congenital anomalies and the use of carbimazole or propylthiouracil in pregnancy.

## 2. Methods

Data on all birth defects reported via the Yellow Card Scheme in association with treatment with carbimazole or propylthiouracil between July 1963 and September 2010 was obtained in “Drug Analysis Prints” from the MHRA [14]. Drug Analysis Prints give a complete listing of all UK spontaneous suspected ADRs reported through the Yellow Card Scheme by healthcare professionals, patients, and the pharmaceutical industry to the MHRA and CHM. They do not present a complete overview of the risks associated with specific medicines, and conclusions on the safety and risks of medicines cannot be made on the information contained in Drug Analysis Prints alone.

## 3. Results

The Drug Analysis Print from the MHRA included 64 reports of birth defects following exposure to antithyroid drugs, reported between 1963 and 2010. Of these, 54 reports came from healthcare professionals, 9 from pharmaceutical companies, and one from a patient. On review, one of the reports was found not to comprise birth defect and was excluded from further analysis. 

**Figure 1:**
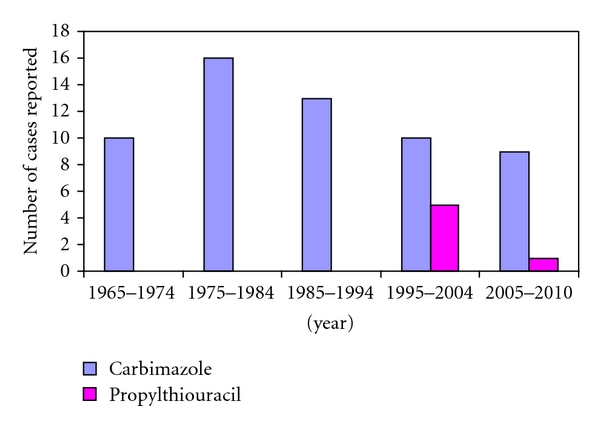
Bar graph showing number of cases with congenital malformations reported following exposure to carbimazole or propylthiouracil by decade from 1965 until 2010.

Figure 1 shows the total numbers of birth defects reported following exposure to carbimazole and propylthiouracil by decade. For carbimazole, there have been 57 cases with a total of 97 congenital anomalies. Three (5%) of these cases (with tracheo-oesophageal fistula, anencephaly, and unspecified congenital heart disease, respectively) have been reported as fatal. For propylthiouracil, only 6 cases with 11 congenital anomalies have been reported, but these have all been within the last 15 years. None of the six cases has been reported as fatal. 

Table 1 describes the type of birth defects reported for carbimazole and propylthiouracil exposure in utero and the number of defects seen in conjunction with other anomalies in the same individual. Two-thirds of the cases with birth defects associated with both carbimazole and propylthiouracil exposure had multiple anomalies in the same individuals. Birth defects associated with carbimazole exposure included aplasia cutis, choanal atresia, tracheo-oesophageal fistula, patent vitellointestinal duct, and dysmorphic facies, which have been previously reported as components of carbimazole embryopathy [9]. The doses of carbimazole used were known for 34 out of 57 cases (60%), and ranged between 5 mg and 60 mg daily (median 15 mg). Similarly, the dose of propylthiouracil, known for 5 out of 6 cases (83%), ranged widely from 50 mg to 350 mg daily (median 50 mg).

## 4. Discussion

Our results support an association between exposure to carbimazole in utero and birth defects. There have been far fewer reports via the Yellow Card Scheme of birth defects related to propylthiouracil exposure, but all the reports related to this drug have been within the last 15 years; this may reflect the fact that historically carbimazole has been the more widely prescribed drug in the UK [15], rather than it its rate of teratogenicity being significantly higher. There has been a 3.5-fold increase in PTU prescription relative to carbimazole since 1981 in the UK [15], which may have unmasked adverse effects that had previously gone unnoticed. Changes in prescribing trends are also reflected by data from the USA, where between 2002 and 2008, propylthiouracil use increased in women of childbearing age [16]. 

The teratogenicity of anti-thyroid drugs remains a source of controversy [17]. Two previous studies comparing carbimazole with propylthiouracil showed no difference in the number of major congenital anomalies seen in babies exposed to these drugs in utero [12, 18]. However, both studies were relatively small. A study from Sweden found 4 reports between 1995–2000 of infants born with oesophageal atresia and omphalocele or choanal atresia, 3 of whom had been exposed to methimazole in the first trimester; there was no association between these anomalies and propylthiouracil [19]. A recent larger case control study which included over 18,000 cases with congenital malformations, 127 of whom were exposed to antithyroid drugs in the first trimester, showed a significant association between exposure to carbimazole/methimazole and choanal atresia or omphalocele [20]. For propylthiouracil, there was a tentative suggestion of an association with situs inversus, renal agenesis, or dysgenesis and cardiac outflow tract malformations although these were not as strong as the associations reported for carbimazole [20]. Consistent with these observations, we found five cases of choanal atresia and four cases of umbilical anomalies associated with carbimazole exposure in our study (Table 1). There were no reports of situs inversus, renal agenesis, or cardiac malformations associated with propylthiouracil exposure (Table 1). 

The nature of congenital malformations seen in our cases is wide ranging, keeping with previous reports of birth defects related to these drugs (Table 1). Several of the congenital malformations associated with carbimazole exposure observed in this study, including aplasia cutis, choanal atresia, tracheo-oesophageal fistula, omphalocele, patent vitellointestinal duct, nipple abnormalities, and dysmorphic facies, have previously been reported in association with carbimazole/methimazole exposure in utero [9]. Furthermore, two-thirds of the anomalies associated with carbimazole exposure occurred with other defects in the same cases, lending further support to an embryopathy as opposed to a single malformation which might have occurred spontaneously irrespective of exposure to teratogens or not. It should be noted that most of the anomalies seen following propylthiouracil exposure also did not occur in isolation, but given the small numbers for propylthiouracil, this should be interpreted with caution.

We acknowledge that there are several limitations to our study. Firstly, true prevalence of birth defects related to carbimazole and propylthiouracil cannot be calculated from the information we have collated. We do not know the total number of births to mothers with Graves' disease over the study period, and we do not have data relating to the types of anti-thyroid drugs prescribed to pregnant women over the study period. In addition, Yellow Card data cannot be used as a reliable indicator of the frequency of suspected ADRs to medicines. The number of reports received via the Yellow Card Scheme does not directly equate to the number of people who suffer adverse reactions to drugs. It is recognised that this scheme is associated with an unknown and variable level of underreporting. The level of ADR reporting may fluctuate between given years due to a variety of reasons, for example, a medicine being new, stimulated interest/publicity, and variations in exposure to the medicine. In this case, there is potential for bias in that prescribers may be more likely to make an association between a congenital anomaly and carbimazole given previous reports of an embryopathy related to the drug which is not the case for propylthiouracil. Similarly, carbimazole and propylthiouracil were introduced to the market at different times, and therefore, reporting bias means that they should not be directly compared.

Secondly, causality cannot be proven. It is important to note that a report of an ADR does not necessarily mean that it was caused by the drug. Many factors have to be taken into account in assessing causal relationships including temporal association, the possible contribution of concomitant medication, and the underlying disease. We do not have information on maternal thyroid function for our cases; this is important, because maternal hyperthyroidism itself is associated with congenital anomalies [2, 3]. Furthermore, in a cohort study of infants of mothers with Graves' disease, the incidence of congenital malformations was significantly higher in infants whose mothers were hyperthyroid in the first trimester compared to those who were euthyroid, with a reported prevalence of 6% and 0.3% in the two groups, respectively [21].

Thirdly, we do not have a complete data on the doses and durations of the carbimazole or propylthiouracil exposure in our cases. We only know doses used for 60% of patients treated with carbimazole and 83% of patients treated with propylthiouracil. 

Nevertheless, the multiple characteristic congenital anomalies we have reported in this study lend support to the teratogenicity of thionamide drugs, in particular carbimazole. This has important clinical implications, and prescribing physicians should be aware of the potential association with congenital anomalies whilst balancing this risk with that of uncontrolled maternal hyperthyroidism in pregnancy.

## 5. Conclusion

The evidence we have described in this study may support a carbimazole embryopathy. There are few birth defects associated with propylthiouracil, but this should be interpreted in the context of higher historical prescription rates for carbimazole.

## Figures and Tables

**Table 1:** Suspected adverse drug reactions of congenital anomalies associated with carbimazole and propylthiouracil received via the UK Yellow Card Scheme.

		Carbimazole			Propylthiouracil		
System	Congenital anomalies*	Number of anomalies (total)	Number with single anomaly	Number with other anomalies	Number of anomalies (total)	Number with single anomaly	Number with other anomalies
Skin	Aplasia cutis	6	2	4	0	0	0
	Other (skin disorder, ulcer)	2	0	2	0	0	0

Respiratory	Choanal atresia	5	2	3	0	0	0
	Tracheo-oesophageal fistula	2	2	0	0	0	0
	Other (neonatal respiratory distress syndrome, respiratory disorder)	2	0	2	0	0	0

Gastrointestinal	Cleft palate	5	3	2	1	1	0
	Omphalocele/umbilical abnormalities	4	2	2	2	0	2
	Patent vitellointestinal duct	1	0	1	0	0	0
	Duodenal atresia	1	1	0	0	0	0
	Anal atresia	1	0	1	0	0	0
	Other (neonatal jaundice and abnormal liver function tests)	2	0	2	0	0	0
	Not specified	3	2	1	0	0	0

Cardiovascular	Septal defects	3	1	2	0	0	0
	Other (Fallot's tetralogy and coarctation of aorta)	2	2	0	0	0	0
	Not specified	1	0	1	0	0	0

Musculoskeletal	Limb/hand/foot malformation	4	1	3	3	1	2
	Not specified	4	3	1	0	0	0

Neurological	Spina bifida	3	1	2	0	0	0
	Hydrocephalus	5	1	4	0	0	0
	Anencephaly	4	4	0	1	0	1
	Hypotonia	2	0	2	0	0	0
	Other (spine malformation and holoprosencephaly)	1	0	1	1	0	1

Renal/urinary tract	Renal aplasia	1	0	1	0	0	0
	Other (urinary tract malformation, epispadias)	1	0	1	1	1	0

Endocrine	Thyroid disorder	1	0	1	0	0	0
	Hypogonadism	1	0	1	0	0	0

Craniofacial	Dysmorphic facies	2	0	2	1	0	1
	Skull malformation	4	1	3	0	0	0
	Ear malformation	3	1	2	0	0	0
	Deafness	4	2	2	0	0	0
	Eye malformation	4	1	3	0	0	0
	Other (nose malformation and teeth malformation)	2	0	2	0	0	0

Others	Nipple/breast anomalies (athelia and hypoplastic nipples)	2	0	2	0	0	0
	Developmental delay	2	0	2	0	0	0
	Autism	0	0	0	1	0	1
	Not specified	7	1	6	0	0	0

Total		**97**	33	64	**11**	3	8

*One Yellow Card report may contain more than one reaction term. Therefore, the total number of reactions is greater than the total number of reports.
